# Exploring the Gut Microbiome in Myasthenia Gravis

**DOI:** 10.3390/nu14081647

**Published:** 2022-04-14

**Authors:** Angel Yun-Kuan Thye, Jodi Woan-Fei Law, Loh Teng-Hern Tan, Sivakumar Thurairajasingam, Kok-Gan Chan, Vengadesh Letchumanan, Learn-Han Lee

**Affiliations:** 1 Novel Bacteria and Drug Discovery Research Group (NBDD), Microbiome and Bioresource Research Strength (MBRS), Jeffrey Cheah School of Medicine and Health Sciences, Monash University Malaysia, Subang Jaya 47500, Malaysia; angelthye.yunkuan@monash.edu (A.Y.-K.T.); jodi.law1@monash.edu (J.W.-F.L.); loh.teng.hern@monash.edu (L.T.-H.T.); 2 Clinical School Johor Bahru, Jeffrey Cheah School of Medicine and Health Sciences, Monash University Malaysia, Johor Bahru 80100, Malaysia; sivakumar.thurairajasingam@monash.edu; 3 Division of Genetics and Molecular Biology, Institute of Biological Sciences, Faculty of Science, University of Malaya, Kuala Lumpur 50603, Malaysia; 4 International Genome Centre, Jiangsu University, Zhenjiang 212013, China

**Keywords:** gut microbiota, myasthenia gravis, autoimmune, acetylcholine, probiotics

## Abstract

The human gut microbiota is vital for maintaining human health in terms of immune system homeostasis. Perturbations in the composition and function of microbiota have been associated with several autoimmune disorders, including myasthenia gravis (MG), a neuromuscular condition associated with varying weakness and rapid fatigue of the skeletal muscles triggered by the host’s antibodies against the acetylcholine receptor (AChR) in the postsynaptic muscle membrane at the neuromuscular junction (NMJ). It is hypothesized that perturbation of the gut microbiota is associated with the pathogenesis of MG. The gut microbiota community profiles are usually generated using 16S rRNA gene sequencing. Compared to healthy individuals, MG participants had an altered gut microbiota’s relative abundance of bacterial taxa, particularly with a drop in Clostridium. The microbial diversity related to MG severity and the overall fecal short-chain fatty acids (SCFAs) were lower in MG subjects. Changes were also found in terms of serum biomarkers and fecal metabolites. A link was found between the bacterial Operational Taxonomic Unit (OTU), some metabolite biomarkers, and MG’s clinical symptoms. There were also variations in microbial and metabolic markers, which, in combination, could be used as an MG diagnostic tool, and interventions via fecal microbiota transplant (FMT) could affect MG development. Probiotics may influence MG by restoring the gut microbiome imbalance, aiding the prevention of MG, and lowering the risk of gut inflammation by normalizing serum biomarkers. Hence, this review will discuss how alterations of gut microbiome composition and function relate to MG and the benefits of gut modulation.

## 1. Introduction

Myasthenia gravis (MG) is an autoimmune condition characterized by muscle weakness induced by autoantibodies binding to the postsynaptic region at the neuromuscular junction (NMJ), impairing the acetylcholine receptor (AChR) function [1]. MG is classified into five main classes and a few subclasses by the Myasthenia Gravis Foundation of America (MGFA) to detect patients with MG showing similar clinical features and severity for better therapy choices [2,3]. There are six subtypes of MG, some in which antibodies bind to different membrane molecules, such as the muscle-specific kinase (MuSK) and lipoprotein receptor-related protein (LRP4), at the postsynaptic NMJ [4,5]. Nonetheless, all types cause defective immunomodulatory signaling leading to generalized or focal muscle weakness and fatigability [6,7]. Other common aspects of MG subgroups are their autoimmune disease mechanism, muscle weakness, and immunosuppressive treatment [8,9]. However, the pathogenesis and therapeutic responses differ, as subtypes are determined by thymus pathology, autoantibody pattern, localization of muscle weakness, sex, and age-onset [5,10].

Epidemiological studies have shown a rise in incidence and prevalence rate for most autoimmune diseases over the past decades [9,11]. The prevalence rate is about 20 per 100,000 [12]. A more recent meta-analysis reported a prevalence rate that ranges from 15 to 179 per million [13]. On the other hand, the overall in-hospital mortality rate in a cohort from the United States was 2.2% [14]. Over the years, there has been an increased prevalence rate, probably due to the availability of improved diagnostic precision and treatments, prolonged survival, and an aging population [15]. The exact etiology of MG is still uncertain, but its development in genetically predisposed patients probably depends upon environmental factors [16].

At present, MG is effectively managed with therapies individualized according to the illness’s age, clinical presentation, pathophysiology, and seriousness [3,5,17,18,19,20,21,22]. Acetylcholinesterase (AChE) inhibitors (neostigmine and pyridostigmine) are used in MG patients’ primary treatment and symptomatic therapy. They increase the level of available acetylcholine at the NMJ without changing the development or outcome of the disease [3,23]. Immunosuppressive medications, including corticosteroids and nonsteroidal immunosuppressive agents, are administrated as continuing immune treatments. The goal here is to prompt near or complete remission [3]. Prednisone is the common corticosteroid used when an AChE inhibitor alone cannot control MG symptoms. In addition, plasmapheresis (PLEX) and intravenous immunoglobulin therapy (IVIg) are rapid short-term immunomodulating treatments that provide improvement of MG within days [3]. Their application is in four circumstances: myasthenia crisis, administrated prior to surgery, patients that are not well-controlled with chronic immunomodulating drugs, and, if needed, to decrease exacerbations before starting corticosteroids [3,24].

Recent discoveries have revealed that the differences in the gut microflora in myasthenia gravis patients could be a promising avenue to explore new therapies and management of MG. The findings suggested that gut microflora may contribute to the disease manifestation and progression. Thus, this review aims to understand how gut microbiome profiles and function changes relate to myasthenia gravis and the benefits of gut modulation in MG patients.

## 2. Myasthenia Gravis—The Pathogenesis, Risk Factors, and Clinical Manifestations

### 2.1. Pathogenesis of MG

The NMJ of skeletal muscles has nerves innervating from the terminal arborization of α-motor neurons of the ventral horns of the spinal cord and brainstem. The synaptic cleft of the NMJ has AChE and supplementary proteins and proteoglycans, whereas on the postsynaptic membrane, AChR is tightly packed above the deep folds. When a nerve action potential (AP) reaches the synaptic bouton, depolarization occurs, opening voltage-gated calcium channels on the presynaptic membrane. Acetylcholine (ACh) is released into the synaptic cleft and diffuses to reach the postsynaptic membrane, triggering end-plate potential (EPP). Later, AChE present in the synaptic cleft will hydrolyze ACh [3].

In an average individual, the EPP generated in the NMJ is much greater compared to the threshold required to a create postsynaptic potential. However, in MG patients, the “safety factor” is reduced. The decrease in AChR molecules causes EPP to decrease, and when EPP falls below the threshold needed to trigger an AP, especially after repetitive activity, symptoms of muscle weakness occur [3,25].

There are four factors contributing to the pathogenesis of MG: First is the effector mechanism of pathogenic anti-AChR antibodies (Abs), which involves complement binding and activation at the NMJ, the antigenic modulation, and the functional AChR block [26]. The second is the role of CD4+ T cells in MG. The synthesis of pathogenic anti-AChR Abs needs activated CD4+ T cells to interact with and stimulate B cells. The third is the role of CD4+ T cell subtypes and cytokines in MG and experimental autoimmune myasthenia gravis (EAMG). Th1 and Th2 are the two subtypes of CD4+ T cells with very different immune response roles. Th1 cells secrete pro-inflammatory cytokines, whereas Th2 cells secrete anti-inflammatory cytokines [3,27]. High levels of anti-AChR Th1 cells are present in the blood of MG patients. They recognize AChR epitopes and induce B cells to produce anti-AChR Abs [3,28,29]. On the other hand, Th2 cells may be protective, but their cytokines contribute to EAMG [26]. Low levels of functioning Treg cells are present in MG patients. Tregs are CD4+ T cells expressing the CD25 marker and Foxp3 transcription factor and are crucial in maintaining self-tolerance. When combined with natural killer T (NKT) cells, it regulates the anti-AChR response. In mouse models, EAMG development is inhibited upon the stimulation of NKT cells [30]. Besides that, antigen-presenting cells (APC) secrete interleukin (IL) 18, which stimulates natural killer (NK) cells to make interferon-gamma (IFN-γ), which enhances Th1 cells, inducing EAMG. Lastly, other autoantigens, anti-MuSK Abs, are present in MG patients lacking anti-AChR Abs. These anti-MuSK Abs affect the maintenance of agrin-dependent AChR clusters at the NMJ, resulting in AChR reduction [3].

### 2.2. Infections as a Risk Factor for MG

The cause and induction of autoimmune diseases have been linked to microorganisms that carry out an unwanted immunological reaction against the self-antigen. This can be seen in an infection causing local inflammation, which increases molecules related to antigen recognition [8,9].

Patients stated that besides stress and surgery, infections also trigger MG events. In Australia, 33.33% of patients reported that infections exacerbated MG, and 50% said the severity was affected by seasonal changes [31]. The effect of disease on MG can be seen in studies from different countries, such as in a population-based study in Spain; almost one-third of MG events were due to infections giving rise to dysphagia and respiratory impairment in MG. In India, it gave rise to MG exacerbations; in a Chinese cohort, it prompted MG relapse [32,33]. Some case studies have shown the connection between certain infections and the simultaneous occurrence of antibodies against NMJ debuting MG-human immunodeficiency virus (HIV), herpes simplex, and hepatitis B and C [31,34,35,36,37]. Although it has been proposed that bystander activation, cryptic antigens, molecular mimicry, epitope spreading, and polyclonal activation are mechanisms inducing MG by viral agents, their effect on the concentrations of specific autoantibodies against AChR, MuSK, and LRP4 during infection is unknown [1,38]. Besides that, the particular microorganism causing MG exacerbations during infection is also unknown, which is likely a broad activation of the immune system [1].

During infections, the genetic predisposition of autoimmune disorders and thymoma are factors aiding the development of MG [38]. Even though genetic predisposition has been verified and linked to human leukocyte antigen (HLA) genes, less than 50% of the total disease risk is due to genetic factors, as specific HLA associations differ for MG subgroups [39,40].

On the other hand, thymoma has AChR Abs associated with MG with a thymus pathology and is responsible for 10% of MG cases, whereas the additional 90% of MG causes are unknown [1,5,41,42]. Although the risk for some diseases is affected by the gut microbiome, there is no data available for the risk of MG and the composition and load of gut bacteria [43]. However, animal models reported that the immune response against microbes could affect the autoreactive immune response via different mechanisms [44]. Nevertheless, infection is a factor that indirectly induces an autoimmune disease and can induce a polyclonal activation of immunoactive cells, but they are yet to be proven for MG etiology [1].

### 2.3. Clinical Manifestations of MG

The disease manifests with varying skeletal muscle weakness, is aggravated when exerted, and improves with rest [10,45]. Although it has overlapping diverse clinical symptoms as with other neurological disorders, the fluctuating feature of MG allows it to be differentiated [46,47]. MG can be categorized into ocular myasthenia gravis (oMG) and generalized myasthenia gravis (gMG) [26]. The oMG symptoms are limited to extrinsic ocular muscles (EOMs), affecting 10% of MG patients. In contrast, in gMG, the most common initial symptom often involves the EOMs, which then spreads involving the bulbar muscle, limb, axial musculature, and respiratory muscles [6,26]. Weakness of the EOMs present as diplopia, blurry vision, and ptosis (unilateral or bilateral). It occurs in 85% of MG patients, in which 50% of them progress to gMG in two years [46]. Around 60% of MG patients presenting bulbar muscle weakness showed fatigable chewing; however, 15% of MG patients showed dysarthria and painless dysphagia as an initial presentation [48,49].

Generally, arms are more affected than the legs in MG patients, and the involvement of facial muscles and the neck extensor and flexor leads to an expressionless face and “dropped head syndrome” [10,50]. Weakness in the respiratory muscles tends to occur only after two years of onset, leading to a life-threatening myasthenic crisis [50].

## 3. Human Gut Microbiome and Its Relation with Human Wellbeing

The human microbiome is defined as ‘the ecological community of commensal, symbiotic, and pathogenic microorganisms that share our body space and have been ignored as determinants of health and disease’ by Joshua Lederberg [51]. It is detailed that 10^10^–10^12^ live microorganisms per gram are found in the human colon [52]. The microbiota in the small and large intestine and the stomach are vital for human health, and they consist mainly of anaerobes living in the large intestine [53]. The human gut microbiome consists of a huge complex symbiotic microbial ecosystem crucial for developing and maintaining the metabolic and immune system homeostasis, impacting human nutrition and the gastrointestinal tract’s function and integrity [54,55,56,57,58,59,60]. It also includes complementing the genes to carry out functions in the human intestine that are not encoded in the human genome [61,62]. Genes encoding functions involved in polysaccharide metabolism, methanogenic pathways for hydrogen gas removal, and enzymes for detoxification of xenobiotics have been identified as enriched functional genetic categories within intestinal microbial communities [62,63].

Dysbiosis refers to alterations in the composition of microbial communities that could influence the host–microbe interaction [64,65]. These changes may, in turn, add to disease susceptibility [65]. Intestinal dysbiosis has been linked to chronic low-grade inflammation [66]; metabolic disorders [67] leading to metabolic syndromes, for example, obesity and diabetes [60,68,69]; infections in the gastrointestinal tract (GIT); irritable bowel syndrome (IBS); and inflammatory bowel disease (IBD) [60,70]. Obesity also relates to dysbiosis and occurs due to the alteration in the microbiota at the phylum level and changes in the representation of bacterial genes and metabolic pathways, where a set of core microbial biomarkers affect the metabolisms [62]. Recently, more evidence on the effect of the microbiota–host interaction on the gut environment and distal organs has become available, with some studies indicating their interaction with the brain and nervous system [71,72,73,74]. There have been studies associating diabetes, cancer, and obesity with alterations to gut microbiome, as well as evidence of the gut microbiome’s implications on the onset of diseases via the “microbiota–gut–brain” (MGB) axis [75,76,77,78,79,80,81,82]. The MGB axis is a communication system made up of intricate loops of neurological reflexes which moderate the coordination between the brain, the intestinal tract, and the endocrine and immune systems involved in maintaining gut function [83,84]. This further proves the function of the gut microbiome as the central regulator of the host immune homeostasis [59,85].

Gut microbes are also responsible for intestinal neuroimmunology [64]. The brain has the most nerve cells, followed by the GIT, which owns an enteric nervous system [86]. Gut microbiota can produce neuroendocrine and neuroactive molecules, for example, histamine, adrenaline, noradrenaline, serotonin, and gamma-aminobutyric acid (GABA), which can communicate through the gut–brain axis [84,87,88]. This, in turn, affects the perception of pain and behavior [64,89]. Furthermore, it is involved in developing neuro-immunological disorders [90]. An experiment involving murine with autoimmune encephalomyelitis (EAE) found that intestinal microbiota caused autoimmunity driven by myelin-specific CD4+ T cells [91]. Treatment with either probiotics or antibodies suppressed the IL-17 production and accumulation of regulatory T cells in secondary lymphoid organs, which improved clinical symptoms and reduced inflammation [92,93,94]. This proposes that probiotics may provide therapeutic benefits for autoimmune diseases by changing the intestinal microbiome [64].

### 3.1. The Relationship between MG and the Gut Microbiome

The gut microbiome could be responsible for triggering diseases in genetically vulnerable individuals [95,96]. MG is an autoimmune disease associated with the dysregulation in the composition and diversity of gut microbiome [97]. Dysregulation of the gut microbiome may be involved in the onset of both central and peripheral autoimmune disorders [98,99]. This is consistent with the recent confirmation demonstrating the relationship of intestinal aberrancies and different autoimmune diseases, for example, Type 1 diabetes [100], IBD [101,102], multiple sclerosis [103,104], and rheumatoid arthritis [103,105], among others. Nonetheless, the pattern of microbial dysbiosis varies for different autoimmune disorders. Thus, microbiota alterations in MG subjects cannot be extrapolated based on other autoimmune diseases. As the microbial diversity and microbial composition of the gut microbiota affects the development of MG, this has resulted in studies using 16S ribosomal RNA (rRNA) gene sequencing and fecal metabolomics being conducted to determine whether the above statement is true and, if so, how it is associated [97].

The gut microbiota is involved in MG development [16]. Alterations to the microbiota affect human physiological functions via the immune system regulation [106]. This is seen in MG disease, where alterations in the gut microbiota affect Foxp3+ CD4+ Treg cells, contributing to MG pathogenesis. MG’s pathogenesis is linked with high levels of circulating AChr Abs. Based on several studies, their production has been linked to Th1, B cells, and Foxp3+ T regulatory (Treg) cells disequilibrium [45,107,108]. The frequency of Foxp3+ CD4+ Treg cells is significantly reduced in MG patients. This has led to more studies focusing on the pathogenesis of MG [109,110,111]. Foxp3+ CD4+ Treg cells are regulatory cells particularly common in the colonic lamina propria [112]. Both Foxp3+ CD4+ Treg cells and the T-cell receptor (TCR) repertoire of Foxp3+ CD4+ Treg cells can be affected by the gut microbiota [113,114,115]. The TCR repertoire plays a role in increasing the Foxp3+ CD4+ Treg cells by recognizing subsets of commensal bacteria, inducing the differentiation of naive CD4+ T cells into antigen-specific Foxp3+ CD4+ Treg cells, resulting in increased Foxp3+ CD4+ Treg cells [114,115]. Foxp3+ CD4+ Treg cells on the other hand, reduce disease severity and progression by affecting the level of autoreactive T cells and suppressing the activity of autoreactive B cells, hence regulating AChR Abs production. Therefore, their function in maintaining self-tolerance and immune homeostasis makes them crucial in preventing MG development [116]. Currently, the interpretation of the pathogenesis of MG is targeted at the inadequate frequency of Foxp3+ CD4+ Treg cells. It is hypothesized that this deficiency in the intestinal bacteria-induced Foxp3+ CD4+ Treg is related to changes in the composition of the gut microbial community [106]. Further details regarding the correlation between probiotics, Foxp3+ CD4+ Treg cells, and MG disease is discussed later in this paper.

### 3.2. Gut Microbiota Composition between HCs and MG Patients

The microbial composition in both healthy controls (HCs) and MG subjects can be obtained using 16S rRNA gene sequencing. In contrast, the functional readout of microbial activity can be obtained from fecal metabolomics [117,118]. We can identify the link between gut microbes and their functional metabolites [118]. In terms of the composition of gut microbes of HCs and MG subjects, Zheng et al. [97] was able to show that a notable difference at the Operational Taxonomic Unit (OTU) level was present based on the 3D principal co-ordinates analysis. Furthermore, control analyses showed that MG subjects’ microbial composition was not notably grouped according to disease subtype, medication/treatment history, or AChR Ab status. Age and gender were not factors involved in clustering HCs and MG subjects. Thus, this suggests that these confounding factors do not significantly affect the global microbial phenotype.

The linear discriminant analysis effect size was also conducted to identify phylotypes behind the differences between HCs and MG subjects. Results were presented that some microbes were abundant in HCs, while some were abundant in MG subjects [106,119]. The human gut consists of a high diversity of bacterial taxa, mainly belonging to Firmicutes and Bacteroidetes [120]. Based on the analysis conducted by Zheng et al. [97], 80 differential OTUs were recognized and held accountable for distinguishing MG subjects from HCs. These 80 OTUs mainly belonged to the phyla Firmicutes (59/80), Bacteroidetes (14/80), and Actinobacteria (3/80). In comparison with the HCs, out of the 80 OTUs recognized by Zheng et al. [97], 34 OTUs belonging to the bacterial taxonomic families (*Bacteroidaceae*, *Lachnospiraceae*, *Prevotellaceae*, and *Veillonellaceae*) increased in abundance, while the remaining 46 OTUs belonging to bacterial families (*Lachnospiraceae*, *Ruminococcaceae*, *Erysipelotrichaceae*, *Clostridiaceae*, and *Peptostreptococcaceae*) decreased in abundance in MG subjects. The results from different studies show that the microbes’ level varied according to different bacterial genera and families.

Furthermore, Moris et al. [119] and Zheng et al. [97] agreed that at the phyla level, Firmicutes are the dominant fecal microbes of both HCs and MG subjects and that the level of Actinobacteria is lower in proportion relative to HCs. Notably, the level of Bacteroidetes, on the other hand, was found to be in a higher proportion in MG subjects, and this result was consistent across three studies [97,106,119]. For instance, Moris et al. [119] reported that there were higher counts (*p* < 0.05) of total bacteria and of the *Desulfovibrio*- and *Bacteroides*-groups based on a quantitative polymerase chain reaction (qPCR) analysis [119]. There are also findings stating that Firmicutes and Bacteroidetes are the main bacterial phyla responsible for microbiota alteration. The Firmicutes/Bacteroidetes (F/B) ratio was lower in MG subjects than HCs. The F/B ratio describes a pro-inflammatory environment. These inflammatory microbiotas damage the intestinal epithelium and prompt the immune response, resulting in an immunological imbalance. Thus, this is consistent with the drop in the F/B ratio in other autoimmune diseases, such as Crohn’s disease and IBD [106,121,122], demonstrating a correlation between the Firmicutes and Bacteroidetes level and some autoimmune disorders.

The linear discriminant analysis effect size conducted by Qiu et al. [106] was able to pinpoint 11 discriminative characteristics (genus level, linear discriminant analyses (LDA) score >2) with an altered relative abundance. In HCs, the bacteria of genera *Clostridium*, *Eubacterium*, *Faecalibacterium*, *Lactobacillus*, etc. were higher in abundance. Conversely, in MG subjects, the bacteria of genera *Streptococcus*, *Parasutterella*, *Escherichia*, etc. were more elevated in abundance. In other words, MG subjects had a significant drop in *Clostridium* and *Lactobacillus* levels, with Clostridium (under the phyla Firmicutes) being the most depleted, with an absolute amount up to three-times less than in HCs. Similarly, Zheng et al. [97] also reported that the level of *Clostridium* (under the phyla Firmicutes) was found to be much greater in the HCs (*p* < 0.001). MG subjects had a reduced abundance of bacteria belonging to *Lachnospiraceae* and *Ruminococcaceae* families from Clostridiales, which are vital for maintaining a healthy gut based on the OTU analysis [97,123,124].

Furthermore, Moris et al. [119] found large inter-individual variability for the *Bifidobacterium* population using ITS profiling, which could be the cause for the lack of statistically significant (*p* < 0.05) differences among the HCs. However, there were some obvious differences, as HCs had high populations of *Bifidobacterium longum* subsp. *Longum,* followed by *Bifidobacterium adolescentis*, whereas MG subjects had high relative proportions of *Bifidobacterium animalis* subsp. *lactis*, *Bifidobacterium breve*, and *Bifidobacterium dentium* [119]. This demonstrates variations in the microbiota profiles and the levels of specific species of bacterium found in both HCs and MG subjects, not just based on a genus level. These *Bifidobacterium* spp. are classified as actinobacteria at the phylum level and are well-known probiotics living in the stomach and intestine. This shows that dysbiosis of specific species of the bacterium does relate to MG (Table 1). In conclusion, the microbial composition of MG subjects was significantly different from HCs, and it had been correlated to a reduced α diversity in MG subjects. This proposes an unusual microbial status, suggesting an association of gut microbiota dysbiosis with MG [97].

#### Possible Mechanisms by Which Some Gut Microbiota May Contribute to MG Development

Some human physiological functions could be affected by changes in the microbial community via the regulation of the immune system. Currently, particular alterations in the microbial composition, specifically of the *Clostridium* spp., have been recorded to influence the amount and TCR repertoire of Foxp3+ CD4+ Treg cells [106]. Clostridia colonizes the mucus layer near the epithelium and affects the intestinal epithelium cells, resulting in a rise in the expression of 2, 3-dioxygenase and transforming growth factor-beta 1 (TGF-β1), which are responsible for encouraging the naive CD4+ T cells to differentiate into antigen-specific colonic Foxp3+ CD4+ Treg cells [112,114,125]. In turn, these differentiated cells curb the production of the anti-AChR auto-antibody and autoreactive B cells, aiding in suppressing the severity of MG [116]. *Clostridium* plays a role in regulating B cells and Foxp3+ CD4+ Treg cells; these Foxp3+ CD4+ Treg cells are responsible for the overproduction of AChR Ab seen in MG subjects. Hence, replacing the reduced level of Clostridia led to a surge in Foxp3+ CD4+ Treg cells, which are essential for MG prevention, and could be a novel strategy against MG [106].

Even though the exact mechanism through which Clostridia regulates the differentiation and activation of immune cells is uncertain, the possible mechanism is via the joint production of short-chain fatty acids (SCFAs) [126,127]. Clostridia are microbes that are recognized to produce SCFAs as the end product of the fermentation of proteins and carbohydrates [128,129]. These SCFAs could affect T cells by regulating their differentiation into Foxp3+ CD4+ Treg cells [130,131]. This is carried out through at least two separate mechanisms. The first mechanism is that the naive CD4+ T cells are exposed to SCFAs, which will increase the acetylation status of histone H3 in the promoter and the conserved non-coding sequence 3 (CNS3) enhancer regions the Foxp3 gene loci, resulting in the Foxp3+ CD4+ Treg cells’ differentiation [132]. The second mechanism is that SCFAs change the phenotype of dendritic cells (DC), inducing retinal dehydrogenase isoform-1 (Raldh1) expression in DCs to increase the production of retinoic acid (RA), causing the differentiation of Foxp3+ CD4+ Treg cells [106,131]. Hence, as a specific microbial composition produces specific microbial metabolites, this could profoundly affect immunity, which possibly affects MG. Clostridia is the primary producer of SCFAs [128]. Based on Qiu et al. and Moris et al., both showed that the commensal microbe-derived SCFA differs in HCs and MG subjects. The overall SCFA obtained from the fecal contents of MG subjects were lower than HCs (*p* < 0.03, Wilcoxon rank-sum test), with propionate and butyrate specifically having a huge drop (*p* < 0.05, Wilcoxon rank-sum test) [106,119]. This is because most of the SCFAs are produced by Clostridia, which are depleted in MG subjects [128]. Propionate and butyrate are the most abundant SCFAs, whose metabolites are vital in regulating the immune system and inflammatory responses [133,134,135]. It is likely that the decrease in Clostridia caused a drop in microbial metabolites- (SCFAs), which is to a certain extent linked to the lower levels of Foxp3+ CD4+ Treg cells, and subsequently resulted in MG disease [106].

**Figure 1:**
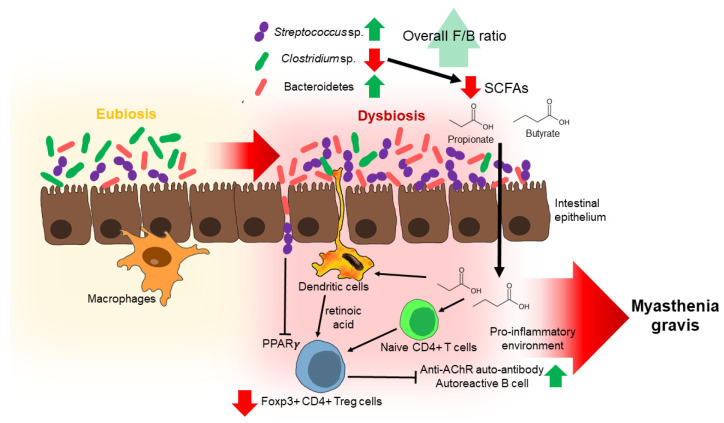
The changes of the gut microbiome as potential mechanisms behind the development of myasthenia gravis (MG).

Besides *Clostridium*, the other genus belonging to the Firmicutes that was shown to have altered levels was *Streptococcus*. A recent study revealed that the level of *Streptococcus* was much higher in MG patients when compared to healthy subjects [106], suggesting that the increased level of *Streptococcus* could have also impacted the tightly regulated host mucosal immune system response. It was reported previously that the commensal bacterium *Streptococcus salivarius* has direct effects on the activation of transcription factors, which have important roles in immune functions [136,137]. In an in vitro study, *Streptococcus salivarius* inhibited the transcriptional activity of peroxisome proliferator-activated receptor (PPARγ) and the subsequent expression of target genes in intestinal epithelial cells, although its exact mechanism is still unclear [137]. PPARγ is a member of a nuclear receptor family that regulates glucose metabolism and lipogenesis as well as immunoregulatory functions in T cells and macrophages [138,139]. PPARγ activation is known to inhibit T-cell activation and inflammatory disease Meanwhile, PPARγ deficiency is associated with an increased disease susceptibility and severity. It was observed that Foxp3+ CD4+ Treg cells decrease in number, while CD4+ IFN-γ cells increase in number with repressed PPARγ, showing its role in Treg survival and effector T cell functions [140]. Therefore, the increased abundance of *Streptococcus* could exhibit an antagonistic effect toward the differentiation of Foxp3+ CD4+ Treg cells, resulting in the Foxp3+ CD4+ Treg cell deficiency as a proposed pathogenesis of MG associated with perturbation of the gut microbiome (Figure 1).

## 4. The Relationship between Gut Microbiome Dysbiosis and Biomarkers in MG Patients

Dysbiosis possibly contributes to serum biomarkers’ variability and promotes chronic inflammation in MG subjects, as they have different gut microbiota profiles [141,142,143,144]. This, in turn, impacts the systemic immune response, as the perturbations of specific gut microbial communities are responsible for biomarkers of autoimmune inflammation [143,144]. Microbe translocation (MT) from an inflamed gut can be used to determine the pro-inflammation status via the serum concentration of lipopolysaccharide (LPS) and endotoxin core antibody immunoglobulin M (EndoCAb-IgM), reflecting MT-correlated immune activation [145]. Previous studies on graft versus host disease [146], IBD/IBS [147], and HIV disease [148], which all involved an increase in lipopolysaccharide secretion soluble CD14 (LPS-sCD14) and EndoCAb-IgM, have described MT as an exclusive pathogenic feature. As commensal flora work synergistically with the intestinal barrier and interact with the innate immune system, changes to the microbial composition enable both microbes and their metabolites to pass through the intestinal barrier, evading the immune intervention, and into the circulation, resulting in immune activation and chronic systemic inflammation [148].

LPS is a crucial outer membrane component of Gram-negative bacteria. It is also a recognized biomarker. LPS has a central role in the host–pathogen interaction, facilitating the process of infection. It binds to cell-surface receptors, for instance, cluster of differentiation 14 (CD14)/toll-like receptor 4 (TLR4)/myeloid differentiation factor 2 (MD2), which is present in various types of host cells, including monocytes, macrophages, B cells, and dendritic cells, and after that, it induces the section of eicosanoids, nitric oxide, and pro-inflammatory cytokines [149]. Furthermore, it has been demonstrated that LPS enhances the immune response to antigens and is a B cell-specific mitogen in mice. For instance, Allman et al. used LPS as an adjuvant to induce an EAMG model and found that LPS-AChR induced mice showed MG-like symptoms and that anti-AChR Abs were produced in the sera, with deposits of IgG2 and C3 at the NMJ [150]. In a study by Rose et al. on the induction of an experimental thyroiditis model using mice, they found that the injection of thyroglobulin together with LPS induced thyroglobulin-specific autoantibodies and lesions in the thyroid [151]. Although some studies also proposed that LPS may be associated with allergies and autoimmune diseases, including experimental autoimmune arthritis [149,152], its role in inducing MG needs further investigation.

MG subjects have different gut microbiota profiles, which could be associated with the variability in serum biomarkers. This led Qiu et al. to assess the gut microbial translocation using biomarkers EndoCAb-IgM and LPS-sCD14 and also assess the dysbiosis-associated chronic system inflammation in MG subjects using other biomarkers involving IL-6 and tumor necrosis factor-alpha (TNF-α). The univariate analyses showed an increase in IL-6, TNF-α, and secretory Immunoglobulin A (SIgA) serum levels and a drop in EndoCAb-IgM and LPS-sCD14 serum levels in MG subjects (*p* < 0.05). The Spearman method was also conducted to find the correlation between the biomarkers and the altered taxa. Qiu et al. possibly suggests that there may be only a slight correlation between the related inflammation and MT. Thus, the rise in systemic inflammation correlates with changes in the microbial community in MG subjects. However, it is possible that it only promotes the expression of inflammatory mediators and not the pathogenesis of MG [106].

## 5. Alterations in Fecal Metabolome of MG Patients

MG is linked with an alteration of the human microbiota, causing an effect on both body function and metabolism and with some microbes associated with the MG severity. Some microbes were linked to AChR Ab, an established biomarker for MG and thymic hyperplasia, affecting the onset of MG. Besides that, alterations in fecal metabolomics also affect MG [97]. As mentioned earlier, fecal metabolomics is carried out to obtain the microbial activity’s functional readout. Zheng et al. found that the fecal metabolic phenotype of HCs was different compared to MG subjects. There were 30 fecal metabolites recognized and held responsible for distinguishing if the subject had MG. Among the 30 metabolites, the level of 16 metabolites reduced in MG subjects, while the level of the other 14 metabolites increased. Then, a functional clustering analysis that was conducted proved the consistency that most of those differentially expressed metabolites had a role in amino acid metabolism (leucine, methylmalonic acid, valine, O-Succinylhomoserine, 5-aminovaleric acid, cysteinylglycine, and D-Glyceric acid), microbial metabolism in diverse environments (xanthine, naphthalene, oxalic acid, catechol, D-Glyceric acid, and 4-nitrophenol), and nucleotide metabolism (cytosine, methylmalonic acid, xanthine, adenine, and oxalic acid), which, in turn, could affect MG occurrence [97].

The alteration in metabolite biomarkers has been correlated with the dysbiosis of the gut microbial OTUs and the clinical symptomatology of MG. The disturbance in the three metabolic pathways mentioned above could potentially be a new complement to the AChR Ab-mediated pathogenesis and diagnosis of MG. Although it is unclear how these metabolic pathways are involved in the onset of MG, there are two hypotheses. The first hypothesis is that the metabolites are associated with the microbial metabolism; thus, the perturbations in the gut microbiome relate to MG. This is consistent with current findings that the fecal metabolome is the functional readout of the gut microbiome. The second hypotheses relate to the metabolism of nucleotides, in which the alterations of cytosine and methylmalonic acid in MG subjects have proposed a link between pyrimidine metabolism and MG. It was also found that MG subjects experience a disturbance in purine metabolism involving xanthine, adenine, and oxalic acid [97]. Thus, it is clear that MG is probably associated with the disturbance in the nucleotide metabolism by modulating oxidative stress, which is a theory supported by earlier studies describing the altered antioxidant status at the protein or metabolite levels [153,154].

## 6. Link between Gut Microbial OTUs with Metabolites and Some Clinical Characteristics of MG

The gut microbial OTUs could also be associated with the metabolites and certain clinical characteristics of MG. Zheng et al. conducted a correlation analysis to explore the relationship between dysbiosis, fecal metabolome, and MG’s clinical symptomatology. There were three findings. Firstly, differential bacterial OTUs were associated with differential metabolites, with 38.75% (31/80) of altered bacterial OTUs having correlations with a range of metabolite biomarkers (r > ±0.35, *p*-value < 0.001). This shows that MG was simultaneously characterized by dysbiosis and the fecal metabolome. Secondly, some gut microbial OTUs were linked to some parameters, including thymic hyperplasia, gender, long-term immune therapies, well-established AChR Abs, and the hamilton anxiety scale (HAMA). Besides that, some OTUs primarily belonging to Lachnospiraceae, Erysipelotrichaceae, and Bacteroidaceae were associated with indicators of MG severity [97].

As mentioned above in the findings from the correlation analysis conducted by Zheng et al., 31 out of 80 microbial OTUs exhibited a link with various metabolite biomarkers; thus, he then conducted a binary regression analysis to detect and quantify the prospective diagnosis ability of the newly found microbial and metabolic biomarkers in MG. The results were that four OTUs, namely Clostridiaceae, Lachnospiraceae, Erysipelotrichaceae, and Bacteroidaceae, and six correlated metabolites, including cytosine, D-Glyceric acid, leucine, N-Acetyltryptophan, oxalic acid, and xanthine, had been causing great deviations between HCs and MG subjects. This led to a huge discovery of combining metabolic biomarkers and microbial markers to discriminate MG subjects from HCs with 100% accuracy compared to diagnosing based on the microbial and metabolic biomarkers separately. Identifying biomarkers for severity is essential, especially for those with a history of a respiratory crisis that could lead to morbidity and mortality [97]. Unlike using AChR Abs as the diagnostic biomarker of MG, which does not reflect MG severity, using the combination of metabolic biomarkers and microbial markers allows for the identification of MG severity; thus, it is a potential diagnostic biomarker that provides significant clinical value [155]. Besides that, a panel of microbes (Clostridiaceae, Lachnospiraceae, Erysipelotrichaceae, and Bacteroidaceae) has been linked to MG severity in terms of a history of respiratory crisis, severity score, and requirement of short-term immune therapies, whereas some metabolites (cytosine, D-Glyceric acid, leucine, N-Acetyltryptophan, oxalic acid, and xanthine) have deviations between HCs and MG subjects. This further implies the potential advantages and strengths of identifying markers for MG severity via analyzing the fecal gut metabolomics [97].

## 7. Insights and Future Perspectives on the Treatment of MG Based on Gut Microbiome Modulation

### 7.1. Probiotics

The diet manipulates and shapes the gut microbiota composition and function [64]. Besides supplements, probiotics are readily available through functional foods and drinks. Probiotics are defined as ‘living microorganisms, which, when administered in adequate amounts, confer health benefits on the host’ by the Food and Agricultural Organization of the United Nations and the World Health Organization [156]. Nobel laureate Elie Metchnikoff introduced the concept of probiotics, and now, probiotics are widely marketed as functional foods or dietary supplements [64,157].

A huge variety of food and drink products contains probiotic strains [158]. Dairy products, including cheese, yogurts, and fermented milk, constitute important probiotic sources in humans, with yoghurt having the highest sales [159,160,161]. Non-dairy products, including cereals, soy-based products, nutrition bars, and juices, can be a way for consumers to obtain probiotics [162,163]. However, it is important to consider the product’s compatibility with the microorganism; safety; efficacy; and the maintenance of its viability via product processing, packaging, and storage conditions. One of the factors affecting the growth and survival of the probiotic is pH, which is the reason soft cheeses are better delivery systems than yoghurt for delivering probiotics to the GIT [164,165,166].

Probiotics influence the composition and function of gut microbial communities via the production of growth substrates or inhibitors, competition of nutrients, and modulation of intestinal immunity [167]. Probiotics also affect the patterns of gene expression. In a recent study, duodenal specimens collected before and after a 6-week intervention period from healthy volunteers taking probiotics showed changes in transcriptional networks involving mucosal biology and immunity [168]. The mechanisms of probiotics include suppression of pathogens, manipulation of intestinal microbial communities, immunomodulation, stimulation of epithelial cell proliferation and differentiation, and fortification of the intestinal barrier [169].

Probiotics use different mechanisms in suppressing bacterial pathogen’s proliferation and virulence. They can produce metabolic compounds or microbial agents that halt other microorganisms’ growth or compete for binding sites and receptors with other intestinal microbes on the intestinal mucosa [170,171,172]. They can also produce a wide range of antimicrobial factors, for instance, bacteriocins and non-peptide compounds, which offer therapeutic alternatives in targeting multidrug-resistant pathogens. For example, in lactobacilli, lactic acid and reuterin are the recognized antimicrobial effectors, with certain strains of *Lactobacillus reuteri* (*L. reuteri*) secreting bacteriocins, affecting both innate and adaptive immunity [64,173,174].

Probiotics can manipulate the intestinal microbial community by inducing the production of β-defensin and Immunoglobulin A (IgA) [169]. In a study where infants were treated with daily supplements of *Lactobacillus casei* subsp. Rhamnosus (LGG), there was a rise in the evenness index in the fecal microbiota, which indicates ecological stability [175]. This increase in ecological stability has been linked to the diversity in microbial communities [176]. Thus, this proves that probiotics do manipulate the intestinal microbial communities and stabilize it.

Probiotics could regulate intestinal immunity and change the responsiveness of the intestinal epithelia and immune cells to microbes in the intestinal lumen [169,177]. The role of probiotics in intestinal immunomodulation involves the upregulation of anti-inflammatory factors and the downregulation of pro-inflammatory factors. In contrast, fortification of the intestinal barrier involves inducing mucin production and maintaining tight junctions [169]. Thus, enhancing intestinal barrier integrity leads to greater immune tolerance and less translocation of pathogens across the intestinal mucosa [178]. Probiotics can also use substrates from the diet to produce secreted soluble factors and metabolites, for instance, vitamins and SCFAs, via signaling pathways such as mitogen-activated protein kinase (MAPK) and nuclear factor kappa B (NFκB) [169,179]. These compounds influence the growth and function of the intestinal epithelium and mucosal immune cells, leading to cytokine and related factors and B cell activating factors production [179]. For example, in gnotobiotic pigs, heat-killed *L. reuteri* 100-23 induces anti-inflammatory cytokine IL-10 production, eliciting an intestinal immune response and regulating the development and recruitment of regulatory T cells to the GIT epithelium [180]. *L. reuteri* can produce soluble factors to inhibit pro-inflammatory cytokine production and signaling of immune cells [181]. Thus, this proves the ability of probiotics in modulating the intestinal immunity and altering the responsiveness of the intestinal epithelia and immune cells to microbes in the intestinal lumen [169,177].

Probiotics have been recommended as a therapeutic and preventive measure in restoring the gut microbiome, and their effects on specific diseases have been studied [119]. Probiotics can increase the functionality of existing microbial communities and introduce advantageous functions into the GIT [64]. Therefore, disease states are possibly related to the changes in core microbial functions [62]. However, more research using controlled human studies is needed to establish the safety and limitations of probiotics, the strain of probiotics, and the dosage required for the greatest efficacy for a particular group of patients. Besides that, the regulatory status of probiotics as food components needs international verification, particularly regarding the safety, efficacy, and validation of a food label’s health claims [158].

### 7.2. Prebiotics

The combined use of prebiotics and probiotics may have a synergistic effect [182]. This combination can improve the survival and implantation of live microbial dietary supplements in the gastrointestinal flora of the host, improve the microbial balance of the GIT, and modify the composition of colonic microflora by selectively stimulating the growth or activating the catabolism of a limited number or one of the health-promoting bacteria in the intestinal tract, leading to the predominance of some of the potentially health-promoting bacteria, particularly, bifidobacteria and lactobacilli [182,183].

A prebiotic is “a nondigestible food ingredient that beneficially affects the host by selectively stimulating the growth and/or activity of one or a limited number of bacteria in the colon” [183]. They can modify the function and composition of the gut microbiota [184]. Gut microbes ferment these prebiotics, and they get their survival energy from degrading indigestible binds of prebiotics [183,185]. Thus, they selectively influence the gut microbiota [186,187]. The two important groups of prebiotics that have advantageous effects on human health are the galacto-oligosaccharides and fructo-oligosaccharides [188]. Currently, inulin-type fructans involving native inulin, synthetic fructooligosaccharides (FOS), and enzymatically hydrolyzed oligofructose or inulin are the only prebiotics with data adequate for possible classification as functional food ingredients [189]. Inulin is defined as a polydisperse carbohydrate material containing largely β-(2-1) fructosyl-fructose links [190]. Both inulin and oligofructose can be found in large amounts in various vegetables and fruits, including leeks, wheat, banana, onion, and garlic [191].

The prebiotic inulin-type fructans have an effect on the GIT, possibly providing a synergistic effect to the use of probiotics. They can avoid digestion in the upper GIT due to the β-configuration of the anomeric C-2 in their fructose monomers, with further evidence that they are not significantly absorbed. Therefore, this allows for the greater survival of bacteria passing through the upper GIT, boosting their effects in the large bowel, and they have been referred to as a colonic food, which is a food acting as a substrate for endogenous bacteria after entering the colon, thus directly supplying the host with metabolic substrates and energy. Many in vivo and in vitro (both microbiological and analytical) studies have supported the proposal that inulin-type fructans are fermented by bacteria colonizing the large bowel and confirming that the end products of fermentation produce SCFAs, including propionic acid, butyric acid, and lactic acid, which have few effects on the body [183,192,193]. For example, SCFAs decrease the colonic pH, leading to alterations in the population and composition of the gut microbiota, affecting acid-sensitive species (Bacteroids) while promoting Firmicutes to produce butyrate (i.e., the butyrogenic effect), whereas propionate influences dendritic cells in the bone marrow and influences T helper 2 cells in the macrophages and airways [192,193,194,195,196,197]. Besides that, peptidoglycan produced from fermentation could stimulate the innate immune system against pathogenic microorganisms [192,198]. On top of that, based on human in vivo studies, this fermentation results in the selective stimulation of growth of the bifidobacteria population. Thus, this makes inulin-type fructans the prototypes of prebiotics [183]. Even though many studies have been conducted on prebiotics’ positive effects on human health, genomic investigations and accurately designed long-term clinical trials need to be carried out to confirm the health claims [188].

### 7.3. Intervention of the Gut Microbiome by Fecal Microbiota Transplantation

The fecal microbiota transplant (FMT) is a translational experimental model that is beneficial in determining if gut microbiome dysbiosis is linked with the development of different diseases [97]. The transplantation of the MG microbiota can affect the outcome of MG. This could be seen in an animal experiment conducted by Zheng et al. using mice under matching immune conditions. The FMT was carried out by colonizing germ-free (GF) mice with an MG microbiome (MMb) or healthy microbiota (HMb) or both MMb and HMb (CMb) and then using classic modeling methods to immunize them [97,199,200]. One month after FMT, an open field test (OFT) was conducted to determine if MMb affects MG-related locomotion. Regardless of the sexes of the mice, the total distance traveled by both MMb and CMb mice dropped more than HMb mice, proving that the colonization of GF mice with MMb, although under identical immune conditions as the HMb colonized mice, resulted in impaired locomotion. Besides that, the level of TNF-α, IFN-γ, and IL-10 in both serum and intestinal tissue were much greater in the MMb group than the HMb group [97]. This is consistent with previous animal and clinical studies showing increased serum TNF-α and IFN-γ [201,202]. As the level of the three cytokines mentioned above in the CMb group were comparable to those in the HMb group, thus, by co-administering HMb into MMb mice, the effect of the increased inflammatory cytokines could be reversed (Table 2) [97]. This is consistent with another finding stating that the interference of the cytokines has demonstrated the ability to alleviate MG severity [203].

#### Alterations in the MG Microbiota of Mice after a Fecal Microbiota Transplant

The 16S rRNA gene sequencing method was also carried out four weeks after FMT, with results showing that compared to HCs, the distinct microbial communities in MG patients were reproducible in FMT, MMb, and HMb mice. The results identified 98 OTUs belonging to Firmicutes (49/98), Bacteroides (34/98), and Actinobacteria (3/98), and these OTUs were able to differentiate between the MMb and HMb groups. Interestingly, they were very similar to the differences seen in the gut microbiome between HCs and MG patients, proposing that the key microbial characteristics seen in MG patients were maintained in MMb mice. Furthermore, 54 of the 98 differential OTUs between MMb and HMb were reversed in the CMb group, in which 16 of the 54 reversed OTUs belonging to *Lachnospiraceae* (7 OTU), *Bacteroidaceae* (4 OTU), and *Ruminococcaceae* (2 OTU) were associated with impaired locomotion ability and interference of fecal metabolomics involving disturbances in the nucleotide metabolism, amino acid metabolism, and microbial metabolism (Table 2) [97].

In a nutshell, the increased level of the inflammatory cytokine, the impairment of locomotion capability, and the disturbed fecal metabolic pathways were seen in the MMb colonized mice, as well as the ability of GF mice colonized with MMb to reproduce the key fecal microbial. The metabolic characteristics of MG patients propose that the gut microbiome could modulate the host metabolism and may be involved in the pathogenesis of MG. Thus, besides providing a better understanding of the pathogenesis of MG, it could provide opportunities to detect possible markers for MG [97].

## 8. Conclusions

**Figure 2:**
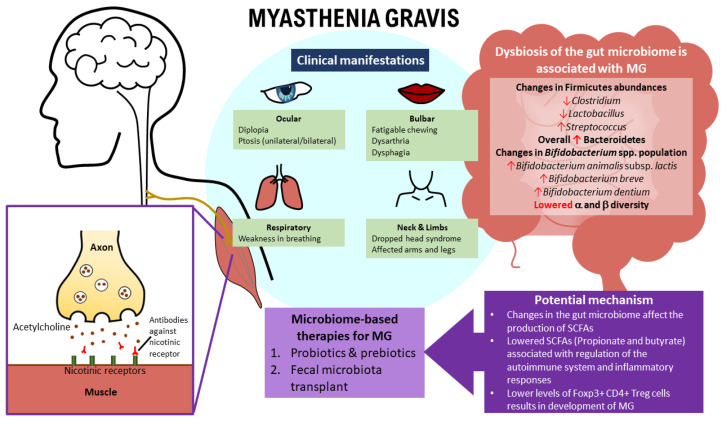
Illustration of how the gut microbiome is associated with the manifestation of myasthenia gravis (MG). It is known that dysbiosis of the gut microbiome could lead to MG’s clinical manifestations. Probiotics, prebiotics, and fecal microbiota transplants are potential microbiome therapies that could be explored and could provide significant benefits to MG patients.

Gut microbiota dysbiosis could be linked with MG (Figure 2). The decrease in the *Clostridium* population could be associated with the imbalance of Foxp3+ CD4+ Treg cells, which are involved in regulating the amount of AChR Abs [116]. Besides this, the fermentation product of Clostridia is SCFAs, and it was found that MG is linked with lower levels of SCFAs, which help regulate the differentiation of Foxp3+CD4+ Treg cells. On the other hand, the higher level of *Streptococcus* in MG subjects may impact PPARγ, which affects transcriptional regulation. However, *Bifidobacterium*, which belongs to the phylum actinobacteria, has a large inter-individuality, indicating that MG is related to specific species of bacterium. Microbial diversity is correlated to MG severity, in which, as the QMG score increases, the α diversity index drops. A total of 30 metabolites were recognized to distinguish MG subjects from HCs, and all are involved in amino acid metabolism, microbial metabolism, and nucleotide metabolism. These changes in metabolite biomarkers correlate with microbial OTUs and the clinical symptomatology of MG, either due to the metabolites’ association with microbial metabolism or the disturbance in nucleotide metabolism involving changes in cytosine and methylmalonic acid or disturbance in xanthine, adenine, and oxalic acid. Moreover, a huge discovery is that the combination of microbial and metabolic biomarkers has been demonstrated to be a potential diagnostic biomarker providing 100% accuracy in discriminating MG subjects from HCs.

Probiotics have been proposed to be a preventive and therapeutic measure against the imbalance in the gut microbiome. They may restore the composition and function of the gut microbiome and stabilize the microbial communities in MG disease, possibly decreasing the severity of the MG disease. A diet consisting of prebiotics and probiotics may be beneficial in improving the survival and implantation of live microbial dietary supplements in the gastrointestinal flora of the host, improving the microbial balance of the GIT, and modifying the composition of colonic microflora by selectively stimulating the growth or activating the catabolism of a limited number or one of the health-promoting bacteria in the intestinal tract, leading to the predominance of some of the potentially health-promoting bacteria, particularly *Bifidobacteria* and *Lactobacilli*.

Nonetheless, there are limitations to the different types of interventions. For instance, a probiotics and prebiotics intervention could be limited to the bacterial strain and the dosage required for the greatest efficacy for a particular group of patients [158,204]. On the other hand, the limitations of FMT would be that the frequency and duration of the FMT may differ between patients, the concerns on the safety and quality check of stool samples, and patients’ acceptance [205]. Significantly, patients’ comorbidities, medications, diet, and lifestyle may also affect the gut microbiota and, hence, the outcome of these interventions. In terms of a future perspective, it is likely to see an increase in MG cases worldwide, but there would be more precise diagnoses and treatments as the aging population increases along with medical comorbidities [15]. For now, extensive population-based data on serological and pathological sub-types of MG within whole populations and an accurate clinical definition over a sufficient time frame in an adequately sized population should be conducted [13]. The dysbiosis of the gut microbiome possibly contributes to disease onset and the progression of MG. Although there is evidence proposing that the gut microbiome plays a role in the pathogenesis of MG, more research needs to be conducted, such as conducting longitudinal studies to collect samples before and after the use of medications to confirm if this correlation is incidental or causal. Furthermore, further research is needed to determine the specific microbial species and their matching metabolite linked to MG, allowing for more specific novel targets for MG treatment. Additional clinical trials are also needed to determine if probiotics can produce the same impact on the human intestinal microbiome and investigate clinical benefits in the host. Lastly, studies need to be performed to detect specific microbial markers correlated with different MG subtypes in recurring MG subjects with varied antibody types.

## Figures and Tables

**Table 1:** Gut microbiome findings in myasthenia gravis (MG) patients.

Studies	Changes in the Gut Microbiome
Zheng et al. [97]	80 differential ^a^ OTUs were recognized and held accountable for distinguishing ^b^ MG subjects from ^c^ HCs. These 80 ^a^ OTUs mainly belonged to the phyla Firmicutes (59/80), Bacteroidetes (14/80), and Actinobacteria (3/80). In comparison with the ^c^ HCs, out of the 80 ^a^ OTUs that were recognized, 34 ^a^ OTUs belonging to the bacterial taxonomic families (*Bacteroidaceae*, *Lachnospiraceae*, *Prevotellaceae*, and *Veillonellaceae*) increased in abundance, while the remaining 46 ^a^ OTUs belonging to bacterial families (*Lachnospiraceae*, *Ruminococcaceae*, *Erysipelotrichaceae*, *Clostridiaceae*, and *Peptostreptococcaceae*) decreased in abundance in ^b^ MG subjects.
	Firmicutes were the dominant fecal microbes of ^c^ HCs and ^b^ MG subjects.
	The level of *Clostridium* (under the phyla Firmicutes) was much greater in ^c^ HCs (*p* < 0.001). ^b^ MG subjects had a reduced abundance of bacteria belonging to *Lachnospiraceae* and *Ruminococcaceae* families from Clostridiales.
	The level of Actinobacteria was lower relative to ^c^ HCs.
	The level of Bacteroidetes was higher in ^b^ MG subjects.
Qiu et al. [106]	In ^c^ HCs, the bacteria of genera *Clostridium*, *Eubacterium*, *Faecalibacterium*, *Lactobacillus* etc. were higher in abundance. Conversely, in ^b^ MG subjects, the bacteria of genera *Streptococcus*, *Parasutterella*, *Escherichia*, etc. were higher in abundance. The level of *Clostridium* (under the phyla Firmicutes) was the most depleted, with an absolute amount up to three-times less than in ^c^ HCs.
	The level of Bacteroidetes was higher in ^b^ MG subjects.
Moris et al. [119]	Firmicutes were the dominant fecal microbes of ^c^ HCs and ^b^ MG subjects.
	The level of Actinobacteria was lower relative to ^c^ HCs.
	^c^ HCs had high populations of *Bifidobacterium longum* subsp. *longum* followed by *Bifidobacterium adolescentis*, whereas ^b^ MG subjects had high relative proportions of *Bifidobacterium animalis* subsp. *lactis*, *Bifidobacterium breve* and *Bifidobacterium dentium*.
	The level of Bacteroidetes was higher in ^b^ MG subjects.
	Higher counts (*p* < 0.05) of total bacteria and the *Desulfovibrio-* and *Bacteroides*-groups based on a ^d^ qPCR analysis.

^a^ OTU: Operational Taxonomic Unit; ^b^ MG: myasthenia gravis; ^c^ HCs: healthy controls; ^d^ qPCR: quantitative polymerase chain reaction.

**Table 2:** Findings of fecal microbiota transplant (FMT) in relation to myasthenia gravis (MG) using mice.

	Findings
Animal study on ^a^ FMT	Using an open field test, 4 weeks after ^a^ FMT [97] - Colonization of ^b^ GF mice with ^c^ MMb resulted in an impaired locomotion ability. - The effect may be reversed by colonizing ^b^ GF mice with both ^c^ MMb and ^d^ HMb. - Levels of ^e^ TNF-α, ^f^ IFN-γ, and ^g^ IL-10 in both serum and intestinal tissue were much greater in the ^c^ MMb group than the ^d^ HMb group. The co-administration of ^d^ HMb could reverse the effects of increased cytokines in ^c^ MMb mice. 16S rRNA gene sequencing, 4 weeks after ^a^ FMT [97] - Distinct microbial communities of MG patients were reproducible in ^a^ FMT, ^c^ MMb, and ^d^ HMb mice. - A total of 98 ^h^ OTUs belonging to Firmicutes (49/98), Bacteroides (34/98), and Actinobacteria (3/98) were identified. - A total of 54 of the 98 differential ^h^ OTUs between ^c^ MMb and ^d^ HMb were reversed in the ^i^ CMb group - A total opf 16 of the 54 reversed ^h^ OTUs belonging to *Lachnospiraceae* (7 ^h^ OTU), *Bacteroidaceae* (4 ^h^ OTU), and *Ruminococcaceae* (2 ^h^ OTU) were associated with impaired locomotion ability and the interference of fecal metabolomics.

^a^ FMT: fecal microbiota transplant; ^b^ GF: germ-free; ^c^ MMb: myasthenia gravis microbiome; ^d^ HMb: healthy microbiota; ^e^ TNF-α: tumor necrosis factor alpha; ^f^ IFN-γ: interferon gamma; ^g^ IL-10: interleukin 10; ^h^ OTU: Operational Taxonomic Unit; ^i^ CMb: both healthy microbiota and myasthenia gravis microbiome.

## Data Availability

Not applicable.
